# Tryptophanyl-tRNA synthetase-1 (WARS-1) depletion and high tryptophan concentration lead to genomic instability in *Caenorhabditis elegans*

**DOI:** 10.1038/s41420-024-01917-4

**Published:** 2024-04-04

**Authors:** Mahmoud Izadi, Tayyiba Akbar Ali, Farah M. Shurrab, Ebrahim Aharpour, Ehsan Pourkarimi

**Affiliations:** 1 Division of Genomics and Translational Medicine, College of Health and Life Sciences, Hamad Bin Khalifa University, Qatar Foundation, Doha, 34110 Qatar; 2 Kevlarr B.V, Nieuwegein, 3436ZZ Netherlands

**Keywords:** Checkpoints, Experimental models of disease, Cancer metabolism, RNAi, Genetics research

## Abstract

The fidelity of translation is ensured by a family of proteins named aminoacyl-tRNA synthetases (ARSs), making them crucial for development and survival. More recently, mutations in the tryptophanyl-tRNA synthetase 1 (*WARS1*) have been linked to various human diseases, from intellectual disability to various types of cancer. To understand the function of WARS1, we investigated the effect of WARS-1 depletion during the mitotic and meiotic cell cycle in the developing germline of *Caenorhabditis elegans* (*C. elegans*) and demonstrated the role of WARS-1 in genome integrity. *wars-1* knockdown results in cell cycle arrest of the mitotically active germ cells. Such mitotic arrest is also associated with canonical DNA damage-induced checkpoint signaling in mitotic and meiotic germ cells. Significantly, such DNA checkpoint activation is associated with the morphological anomalies in chromatin structures that are the hallmarks of genome instability, such as the formation of chromatin bridges, micronuclei, and chromatin buds. We demonstrated that knocking down *wars-1* results in an elevation of the intracellular concentration of tryptophan and its catabolites, a surprising finding emphasizing the impact of cellular amino acid availability and organismal/individual dietary uptake on genome integrity. Our result demonstrates that exposing *C. elegans* to a high tryptophan dosage leads to DNA damage checkpoint activation and a significant increase in the tryptophan metabolites. Targeting tryptophan catabolism, the least utilized amino acid in nature, can be important in developing new cancer therapeutic approaches. All in all, we have strong evidence that knocking down *wars-1* results in defects in genomic integrity.

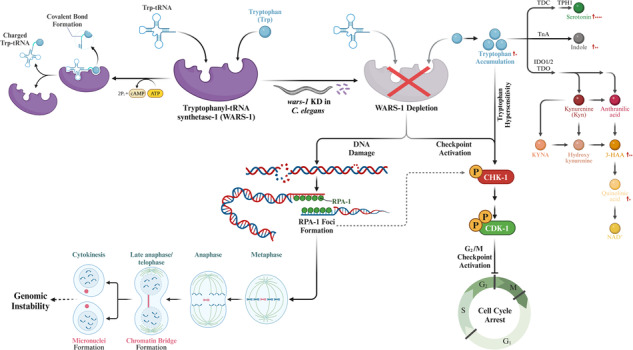

## Introduction

Regulation of protein translation is central to cellular growth and differentiation. mRNA translation occurs in accordance with cell cycle progression. Improper translation or the lack of translational fidelity is associated with multiple human diseases, including cancer and neurodegenerations. In contrast to the replication error rate of nearly one mistake per 100 million to 10 billion bases, mRNA translation occurs at a much lower precision of one amino acid per 1000 to 10,000 [1–3]. Improper protein translation and metabolism are often seen in various types of cancer [4]. The rapid proliferation of tumor cells imposes an unusually high mRNA translational demand on cancer cells, which is accomplished by altering translational regulators. Therefore, alteration of the translational program is associated with malignancies [5], and the specific inhibition of protein synthesis provides an “Achilles heel” for cancer cells, potentially aiding patient survival.

Translational fidelity is ensured by a group of proteins collectively called aminoacyl-tRNA synthetases (ARSs), which are responsible for the loading and aminoacylation of the correct amino acids to their cognate tRNA [6]. ARSs family members are considered housekeeping genes that only function during mRNA translation. However, recent studies have demonstrated their role in inflammation, angiogenesis, and malignancy [7, 8]. Each amino acid has its given ARS protein to ensure the fidelity of the tRNA/amino acid complex as well as the accuracy of mRNA translation in the initial steps [9]. Besides the highly specific aminoacylation domain, ARSs proteins have an evolutionarily conserved editing domain that cleaves mischarged amino acids from tRNA. Defects in the editing domain lead to alterations of the amino acid sequence during translation, which is linked to protein misfolding and ensuing cell death [10–12]. Editing-defective mutants of the *E. coli* isoleucyl-tRNA synthetase suffer from growth retardation under various conditions [11]. Phenotypes associated with the deficiency for tRNA loading are not limited to general growth retardation but can affect specific cell types. Mutation of alanyl-tRNA synthases in mice causes neuronal degeneration of cerebellar Purkinje cells [12]. More recently, a study on tRNA synthetases in zebrafish presented convincing evidence that the transient expression of a valyl-tRNA defective for editing causes a p53-dependent DNA damage response [13].

Emerging genomics and proteomics studies in the past decade clearly link mutations or aberrant expression of various ARS genes to over 50 human diseases, such as neuropathies, hearing loss, microcephaly, and cancer [8, 14]. Mutations of the tryptophanyl-tRNA Synthetase 1 (*WARS1*) have been associated with various human diseases, ranging from intellectual disabilities to cancer and metastasis [15, 16]. More recently, deletions and mutations of *WARS1* have been associated with skin cutaneous melanoma (SKCM), cervical squamous carcinoma, and colorectal cancer [17]. In addition, a high mRNA level of *WARS1* increases the prognosis of SKCM patients [17]. Nonetheless, despite their importance in protein translation, the pathology associated with ARSs deficiency remains to be understood.

Tryptophan (Trp) is mainly degraded via the Kynurenine pathway, leading to the production of divergent metabolites, namely kynurenines. The tryptophan/kynurenine ratio is highly controlled intracellularly, and their imbalanced ratio has been reported in various diseases, including cancer, autoimmune diseases, inflammation, and neuropsychiatric disorders [18–21].

**Fig. 1:**
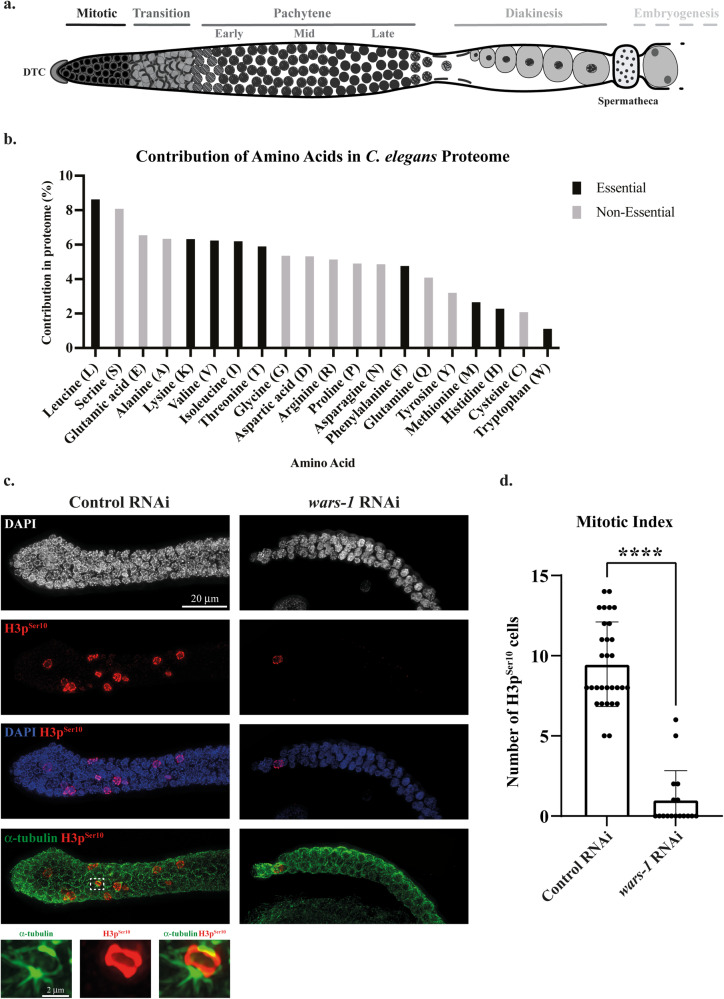
WARS-1 depletion reduces the mitotic index in the *C. elegans* germline. **a** Schematic representation of a hermaphrodite germline. Each adult hermaphrodite worm has two distinct germlines. These germlines are characterized by various stages of mitotic and meiotic cell divisions. The central component of this system is the Distal Tip Cell (DTC), which serves as the stem cell niche responsible for generating all germ cells in the germline. As germ cells migrate from the DTC towards the vulva, they first engage in mitotic proliferation within the mitotic region. Subsequently, they transition through a zone leading into stages of pachytene and diakinesis. Eventually, the mature oocytes pass the spermatheca, where fertilization occurs. The initial phases of embryogenesis then take place before the embryos are laid. **b** Contribution of amino acids in *C. elegans* proteome. Tryptophan is the least used amino acid in the *C. elegans* proteome, as shown in the chart. **c** Reduced Mitotic index upon *wars-1* knockdown (KD). Immunostaining of isolated germline using an antibody against Phosphorylation of histone H3 at Serine 10 (H3p^Ser10^) in wild type and upon *wars-1* RNAi. The bottom row shows a zoom-in of the dashed box showing a metaphase plate with the maximum condensation of chromatin and H3p^Ser10^ intensity in control. **d** Quantification of H3p^Ser10^ positive cells. Number of H3p^Ser10^ positive cells in the control RNAi and *wars-1* RNAi treated worms. Knocking down *wars-1* significantly reduced the mitotic index (****P < 0.0001, *n* > 17). The F-test to compare variances resulted in non-significant differences (*P* = 0.1311). An unpaired t-test was used to compare the differences between the means of the two groups. The error bars represent the standard deviation. The scale bar in panel c represents 20 μm except for the scale bar in the zoomed-in section, which corresponds to 2 μm.

Previously, we reported an association between WARS-1 depletion and defects in germ cell development and mitotic cell cycle progression in the *Caenorhabditis elegans* (*C. elegans*) germline [22]. Our previous observation aligns with a human *WARS1* loss-of-function mutation associated with microcephaly [22]. *wars-1* KD results in sterility and a dramatic reduction of germ cells in various regions, especially in the mitotic zone of the *C. elegans* germline [22]. Germline exhibits distal–proximal polarity, with the mitotically dividing cells at the distal end and the meiotic cells at the proximal region. The germline contains various stages of differentiating germ cells organized in a gradient of differentiation, starting with proliferative cells near the single distal tip cell (DTC), cells entering early meiosis as part of the so-called transition zone, followed by a large compartment of cells residing in the pachytene stage, and finally, cells that increase in size and gradually mature into oocytes (Fig. 1a). In the current study, we aimed to investigate the effect of WARS-1 depletion on cell cycle progression using the *C. elegans* germline system.

## Results

### Tryptophan has the least contribution to the *C. elegans* proteome

There is a long-lasting textbook belief that tryptophan is nature’s least-used amino acid [23]. To test if this is also the case in *C. elegans*, we generated a Java-based program to analyze the amino acid composition of the *C. elegans* proteome. Our result indicated that Trp is indeed the least-used amino acid, with an average of nearly 1.11% of the total amino acid composition in *C. elegans* proteome (Fig. 1b). Surprisingly, only 5% of the free Trp is utilized in protein translation, and the remaining are degraded through stepwise biochemical reactions to various active metabolites, ending in the formation of nicotinamide adenine dinucleotide (NAD) that is involved in redox reactions [24]. Given the universally low composition of Trp in any given proteome, we decided to analyze the link between Trp usage and protein function. We, therefore, analyzed the gene ontology (GO) of proteins composed of various percentages of Trp (3% (twice the average) and above and less than 0.6% (half the average)). Comparing the GO of proteins with the highest usage of Trp to the one of the least showed that Trp is indeed enriched in proteins associated with membranous compartments, while proteins lacking or have the least use of Trp are related to various cellular functions and are predominantly nuclear or cytoplasmic (Sup. Figure S1). Given the complex Trp structure, its enrichment in membrane structure is not surprising. The presence of the indole ring and its potential interaction with other cationic residues, such as Lysin, make Trp an ideal amphiphilic residue, serving at the hydrophobic/hydrophilic boundaries in membrane-associated proteins. This is in accordance with several investigations showing Trp is enriched at the lipid bilayer, stabilizing trans-membrane-spanning proteins [25, 26].

### *wars-1* KD leads to decreased mitotic index

To knock down *wars-1* in *C. elegans*, we generated an RNAi construct against *wars-1* that effectively downregulated its expression (Sup. Figure S2, *P* < 0.0001) [22]. To further characterize the defect in the mitotic cell cycle, we compared the mitotic index of the *wars-1* RNAi germline with that of the wild-type. To this end, we immunostained the extracted germline using an antibody against phosphorylation of Histone H3 at Serine 10 (H3p^Ser10^), which is a canonical marker for mitosis progression [27]. Our immunostaining showed that at any given time in the wild-type worms, there were 10–15 germ cells undergoing mitosis and hence positive for H3p^Ser10^, but significantly reduced upon *wars-1* KD (Fig. 1c, *P* < 0.0001), indicating that germ cells of *wars-1* KD might be arrested during the cell cycle (Figs. 1c, d, Sup. Movie S1–S6).

### *wars-1* KD induces cell cycle arrest

To test whether the reduction in the mitotic index upon *wars-1* KD is associated with cell cycle arrest at the S phase, we analyzed the size of the cell’s nuclei upon WARS-1 depletion. Comparing the nuclei diameter between *wars-1* KD and control showed no significant differences (*P* = 0.802), implying that the mitotic cells of *wars-1* KD were not arrested at the S phase (Sup. Figure S3).

**Fig. 2:**
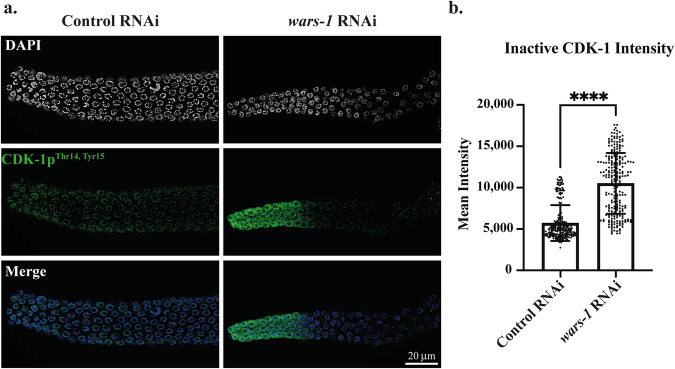
Increased intensity of inactive CDK-1 upon *wars-1* KD. **a** Representative images of fluorescent microscopy. Immunostaining of isolated germlines using an antibody against CDK-1 phosphorylation at Threonine 14 and Tyrosine 15 (CDK-1p^Thr14, Tyr15^) in control and upon knocking down *wars-1*. **b** Quantification of CDK-1p^Thr14, Tyr15^ intensity. Comparing the mean intensity of the CDK-1p^Thr14, Tyr15^ positive cells in the control RNAi and *wars-1* RNAi shows a significant increase in signal intensity of CDK-1p^Thr14, Tyr15^ upon *wars-1* KD (****P < 0.0001). As the F-test to compare the variances showed significantly different variances (*P* < 0.0001), an unpaired two-tailed t-test with Welch’s correction was used to compare the two groups. The experiment was done on at least 4 biological replicates, and more than 230 germ cells per condition were analyzed. The error bars represent the standard deviation. The scale bar shown in panel a denotes 20 μm.

To test if germ cells are arrested at the G2/M transition, we stained the extracted germline with the antibody against cyclin-dependent kinase 1 phosphorylated at Threonine 14 and Tyrosine 15 (CDK-1p^Thr14, Tyr15^). Comparing *wars-1* KD germ cells with the control RNAi revealed that nearly 100% of mitotic cells exhibited hyperphosphorylation of CDK-1p^Thr14, Tyr15^ in mitotic germ cells, indicating that these cells were arrested at the G2/M phase of mitosis (Fig. 2a, Sup. Movie S7–S10). Comparing the mean intensity of the CDK-1 phosphorylation signal of the individual *wars-1* KD mitotic cells with that of the control revealed a significant increase in the CDK-1p^Thr14, Tyr15^ signal (*P* < 0.0001, Fig. 2b).

### Knocking down *wars-1* leads to activation of the DNA damage checkpoint

**Fig. 3:**
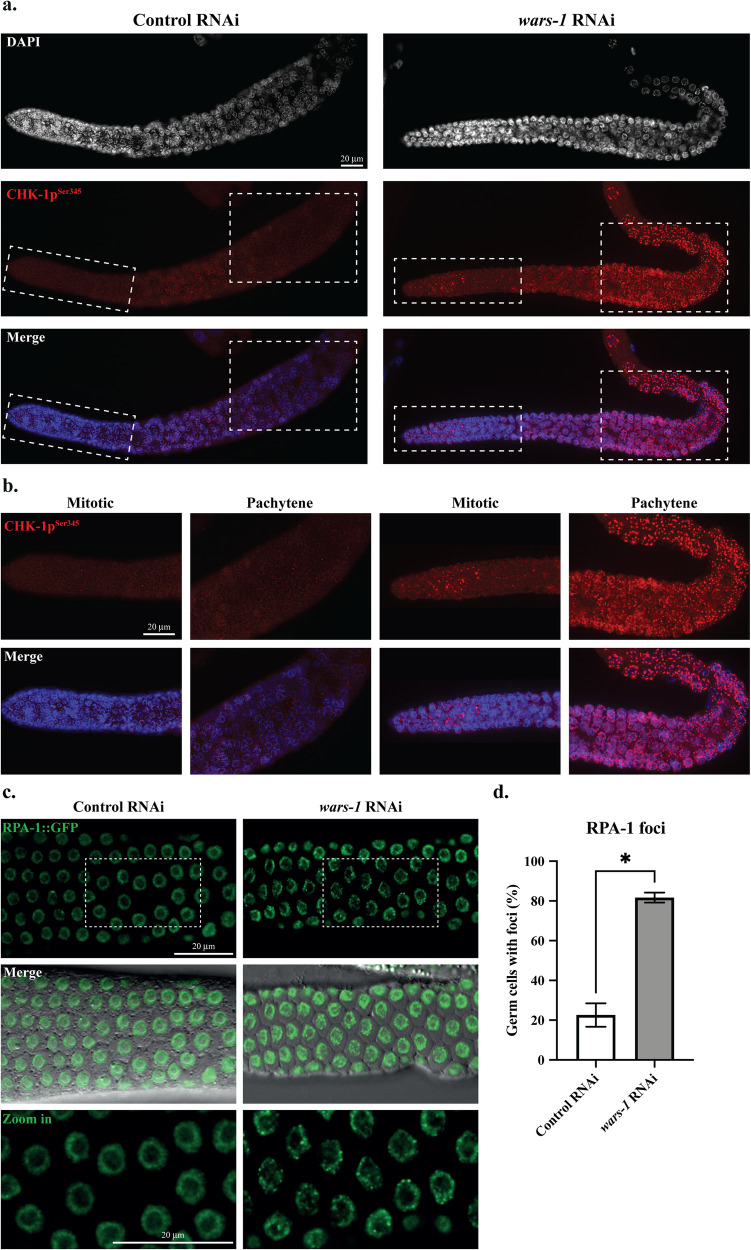
*wars-1* KD induces DNA damage. **a** *wars-*1 KD activates checkpoint signaling through CHK-1p^Ser345^. Upon knocking down *wars-1* (right column), CHK-1 phosphorylation at Serine 345 (CHK-1p^Ser345^) was increased. **b** Zoom-in of the dashed boxes in part a displays the CHK-1p^Ser345^ foci in *wars-1* KD compared to control RNAi both in mitotic and pachytene regions. **c** Replication protein A (RPA) foci formation upon *wars-1* KD. Microscopic images of extracted germlines from SSM473 (*rpa-1::gfp*) worms treated with *wars-1* show increased RPA-1::GFP foci formation compared to the control RNAi. The bottom row shows the zoom-in of the dashed squares in the first row, presenting the remarkedly higher number of foci-positive germ cells. **d** Quantification of RPA-1 foci. The percentage of RPA-1 foci-positive cells in the germline of *wars-1* KD was significantly higher than the control (*P* = 0.0268). An unpaired two-tailed t-test with Welch’s correction was used to compare the two groups. The error bars indicate the standard deviation. For each condition, at least 90 germ cells were counted and analyzed for RPA-1 foci. Scale bars shown in each panel represent 20 μm. (*P < 0.05).

Next, to check whether CDK-1 phospho-inactivation arises from genomic instability and checkpoint activation, we investigated CHK-1 activity upon *wars-1* KD. To this end, we stained the germline of *wars-1* KD animals using an antibody against phosphorylated human checkpoint kinase 1 at Serine 345 (CHK-1p^Ser345^) that cross-reacts with the corresponding phosphorylated Serine of the *C. elegans* CHK-1 [28]. While CHK-1 was not phospho-activated in control RNAi, CHK-1 phosphorylation became apparent in the germ cell nuclei of the mitotic zone, transition, and more strongly at the pachytene upon *wars-1* KD (Figs. 3a, b, Sup. Movie S11–S14).

To test if the effect of the *wars-1* KD on cell cycle progression and checkpoint activation is not a general cellular response to improper protein translation, we treated wild-type worms with cycloheximide (CHX) to inhibit the translation. It is well-established that CHX blocks ribosomal activity and translation in *C. elegans* [29–31]. Immunostaining of the germline treated with CHX for 6 h using anti-CDK-1p^Thr14, Tyr15^, and anti-CHK-1p^Ser345^ antibodies did not show any difference with that to the non-treated control germlines (Sup. Figure S4). Additionally, to rule out that mitotic cell cycle arrest and activation of the canonical checkpoint pathway is not a universal response to the lack of amino acid loading to tRNAs, we systematically knocked down all members of the cytoplasmic ARSs protein family and analyzed the status of the CDK-1 and CHK-1 using immunostaining. In essence, 5 (EARS-1, GARS-1, IARS-1, KARS-1, PARS-1) out of the 20 ARSs members had severe defects in their germline development, leading to a complete lack of germline, and therefore, were not immune stained. (Sup. Table S1). Our anti-CDK-1p^Thr14,Tyr15^, and anti-CHK-1p^Ser345^ immunostaining of all the knockdown of the ARSs protein family members showed no difference with the control RNAi demonstrating the cell cycle arrest phenotype and checkpoint activation is specific to WARS-1 depletion (Sup. Figure S5).

To further characterize if *wars-1* KD leads to genomic instability and DNA damage response, we evaluated the accumulation of RPA-1 in *wars-1* RNAi worms. Interestingly, *wars-1* KD in the RPA-1::GFP expressing worms significantly increased the percentage of RPA-1 foci-positive germ cell nuclei, especially in the pachytene region of the germline (*P* = 0.0268) compared to the control RNAi (Fig. 3c, d). Also, the number of RPA-1 foci per cell was increased in the *wars-1* KD germline.

### *wars-1* KD results in the formation of micronuclei at the pachytene stage of the germline

**Fig. 4:**
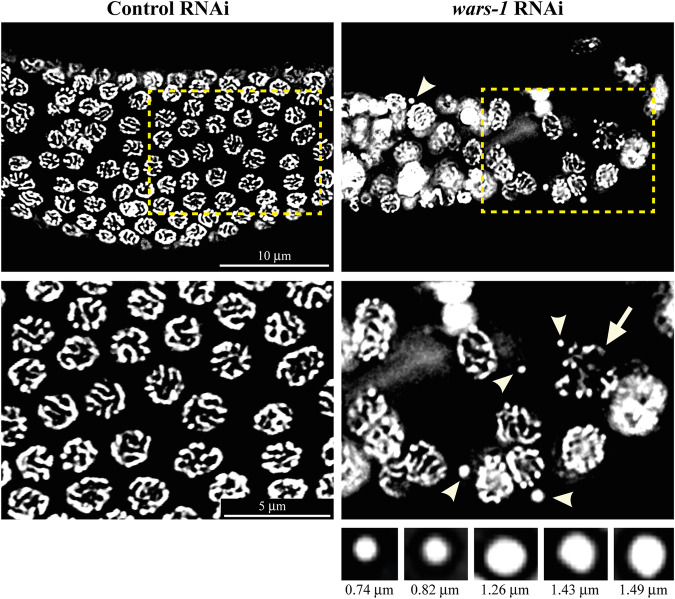
Micronuclei formation upon *wars-1* KD. Knocking down *wars-1* results in the formation of micronuclei and fragmented chromatin, indicating a massive genotoxic insult. The second row is the zoom-in of the dashed boxes in the top row. Micronuclei formed upon *wars-1* KD are of various sizes, ranging from 0.74 to 1.49 μm in diameter (Shown under the *wars-1* panel). Arrowheads point to the micronuclei, while the arrow indicates fragmented and frayed-looking chromosomes. The scale bar in the top row represents 10 μm, and the bar in the second row is equal to 5 μm.

To test if the depletion of WARS-1 during germline development can lead to genotoxic events, such as chromosomal anomalies in both mitotic and meiotic cells, we stained the chromatin of the germ cells using DAPI. Surprisingly, we observed various morphological features associated with chromosomal anomalies, such as micronuclei (MN) and fragmented and often frayed-looking chromosomes at the late pachytene cells (Fig. 4). We compared the size of the micronuclear structures observed upon *wars-1* RNAi by measuring the diameter of the MN at its widest. As expected, the MNs at various pachytene cells exhibited various sizes ranging from 0.74 to 1.49 μm, indicating the stochastic event of chromosomal loss during the cell cycle.

### *wars-1* KD results in the formation of chromatin bridges and micronuclei in the developing embryos

**Fig. 5:**
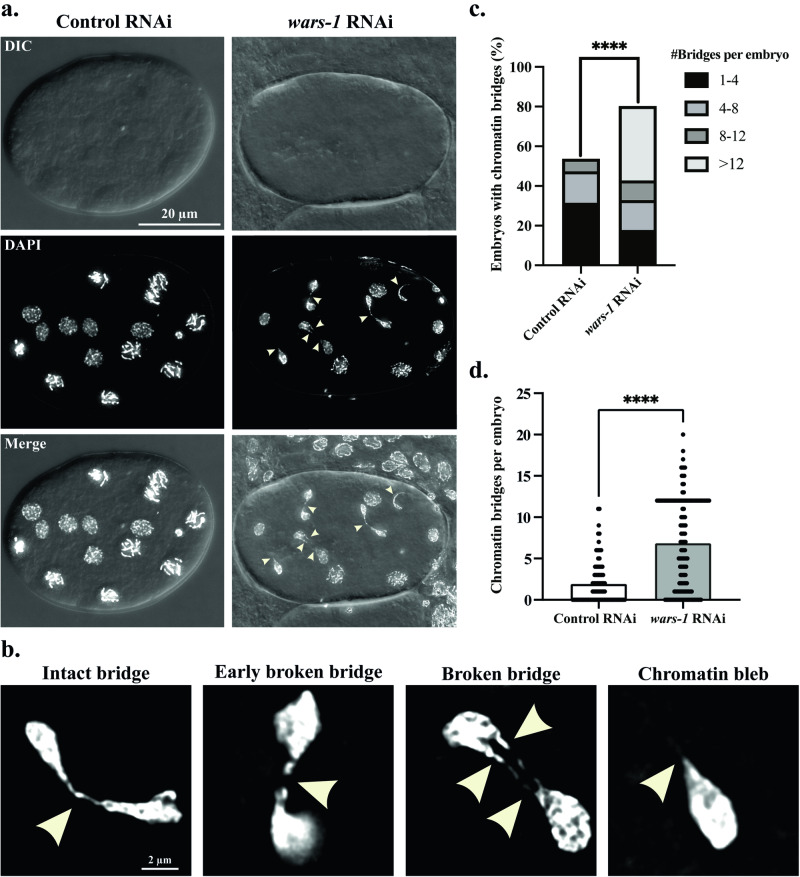
Formation of chromatin bridges upon *wars-1* KD. **a** Knocking down *wars-1* results in the formation of chromatin bridges in the developing embryos. Arrowheads showing chromatin threads between dividing nuclei. Each image represents a projection of 0.3 μm Z stacks. **b** Zoom in on four different stages of chromatin bridges; from left to right, different stages of bridge breakage/resolution are shown. **c** Increased chromatin bridges upon *wars-1* **KD**. Knocking down *wars-1* significantly increased the percentage of embryos with chromatin Bridges (****P < 0.0001). **d** Increased number of chromatin bridges per embryo upon *wars-1* KD. Knocking down *wars-*1 significantly increased the number of embryos with chromatin bridges (****P < 0.0001). As the F-test to compare the variances showed significantly different variances (*P* < 0.0001), an unpaired two-tailed t-test with Welch’s correction was used to compare the two groups. The scale bars in panels a and b represent 20 μm and 2 μm, respectively. For each condition (*wars-1* RNAi and control RNAi), more than 90 embryos were analyzed.

**Fig. 6:**
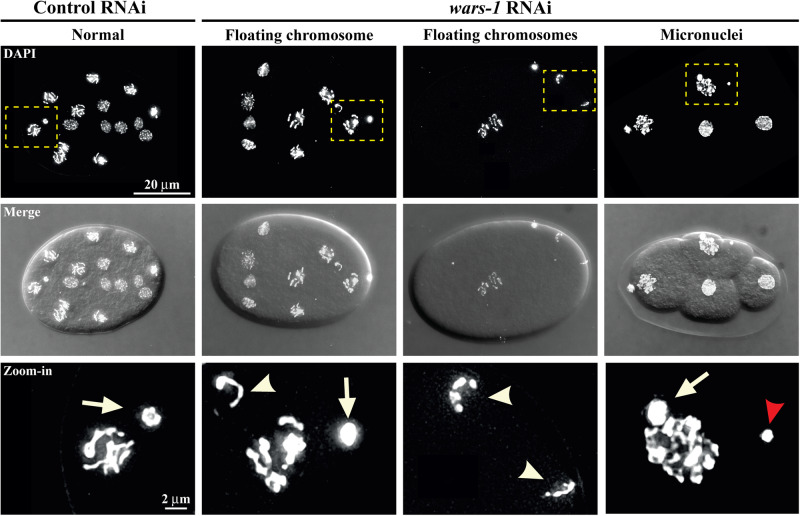
Formation of micronuclei and fragmented chromosomes in embryos upon *wars-1* KD. Upon WARS-1 depletion, fragmented, floating chromosomes and micronuclei were observed in developing embryos, indicating chromatin instability. The bottom row zooms into the squared boxes in the top row. Arrows indicate the polar bodies, while the arrowheads point to floating chromosomes. The red arrowhead indicates a micronucleus in a developing embryo. The scale bar in the first row represents 20 μm, while the scale bar in the zoom-in row indicates 2 μm. Each experiment was performed at least six times, and the representative images were used in this figure.

As mentioned previously, *wars-1* KD in the early first larval (L1) stage of *C. elegans* development leads to a nearly complete sterility [22]. To further analyze the effect of WARS-1 on the genome stability of mitotic cells throughout embryogenesis, we reduced RNAi efficiency by treating third larval stage (L3) worms to *wars-1* RNAi. This ensured the worms developed into hermaphrodite adults with only a partial decrease in fertility, and few of their fertilized oocytes developed into early to mid-stage embryos. Surprisingly, over 80% of developing embryos of the partial RNAi-treated worms exhibited chromatin bridges and aberrant chromosome structures compared to 50% of naturally occurring bridges in the wild-type embryos (Fig. 5a–c). In the *wars-1* KD embryos, different stages of chromatin bridges were detected, including intact bridges, early stages of breakage, and completely broken bridges, as well as the chromatin bleb/bud structures resembling the remnant of a broken/ resolved chromatin bridge (Fig. 5b). In addition, the number of nuclei containing chromatin bridges per embryo significantly increased upon *wars-1* KD from an average of 1.904 to 6.842 (Fig. 5d, *P* < 0.0001). It has been reported that in higher organisms, many cells enter mitosis with incomplete DNA replication, even in the absence of DNA damage [32]. Surprisingly, in normal human cells, ultra-fine chromatin bridges have been reported during mitosis. In nearly 20–30% of cells in late anaphase, ultrafine chromatin has been observed with no apparent sign of increased aneuploidy or DNA damage-induced apoptosis [33]. This may explain the surprisingly and relatively high (50%) chromatin bridge frequency that we observed in the control RNAi. The formation of chromatin bridges also indicates the accumulation of unrepaired DNA breaks or excessive DNA damage during the mitotic and meiotic cell cycle upon WARS-1 depletion. Some of the chromatin bridges that are formed upon WARS-1 depletion resulted in the formation of micronuclear structures in the developing embryos, while we could never observe micronuclear structures in the wild-type embryos (Fig. 6). Interestingly, we have also observed a chromatin loss during embryogenesis upon *wars-1* RNAi (Fig. 6).

### Increased levels of tryptophan lead to toxicity and activation of the DNA damage response

**Fig. 7:**
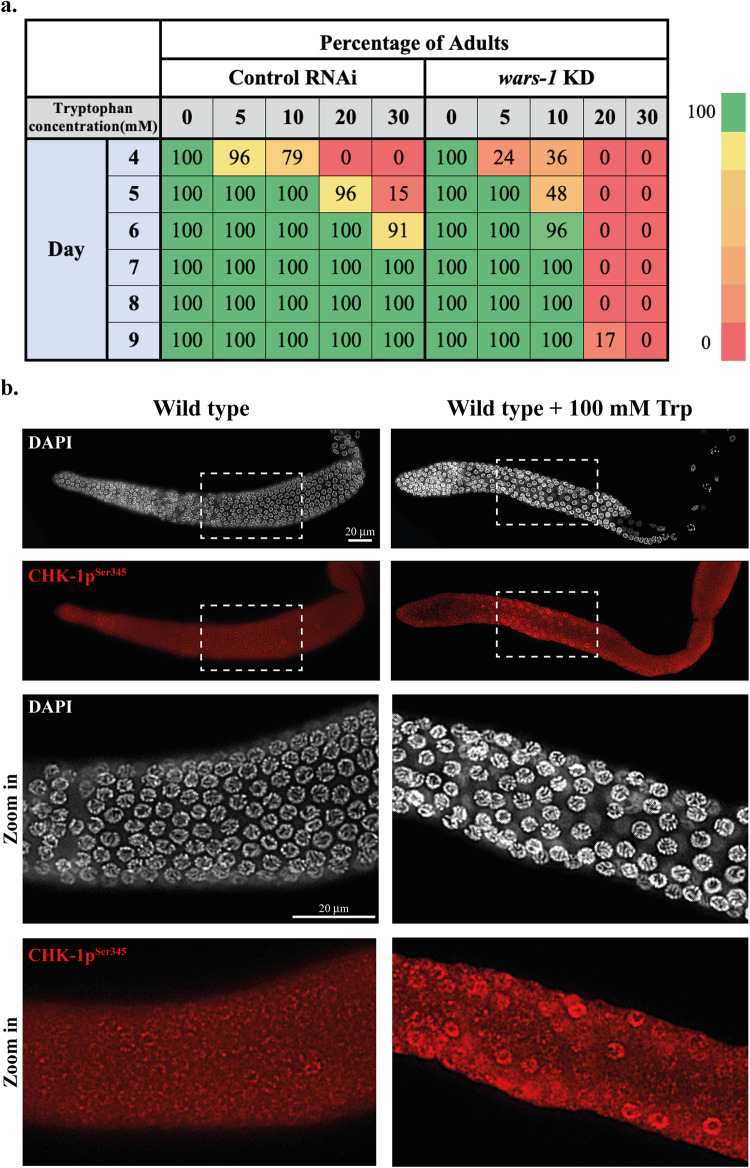
Increased tryptophan concentration lead to toxicity and activation of the DNA damage response. **a** Tryptophan hypersensitivity upon *wars-1* KD. Depletion of WARS-1 leads to the hypersensitivity of worms to the increased concentrations of tryptophan (Trp), shown by reduced percentages of adulthood. At increased concentrations of Trp (>10 mM), worms arrest at the earlier stages of development. (Day 1 is considered the first day of larval development, and worms were observed for nine days. For each condition (*wars-1* RNAi and control RNAi) and concentration (0–30 mM), over 20 worms were analyzed. The experiment was repeated at least 3 times. The color gradient bar on the right side indicates the percentages of worms, with green and red showing 100% and 0%, respectively. **b** Checkpoint activation following acute tryptophan exposure. Exposure to Trp at a higher dosage than physiologic levels for 24 h induced checkpoint activity, as shown by phosphorylation of Checkpoint kinase 1 at serine 345 (CHK-1p^Ser345^). Zooming into the pachytene zone (marked by the dashed squares) indicates the increase in CHK-1 activity upon acute exposure to high levels of Trp (bottom rows). The scale bars represent 20 μm. The experiment was repeated at least three times.

Finding excessive chromatin bridges implies that the improper translation due to a lack of tryptophan loading to its cognate tRNA leads to genome instability. This may be due to the increased unloaded Trp that could be hazardous to cellular function. We, therefore, tested if *wars-1* KD developing worms are hypersensitive to high Trp concentrations. To this end, we treated the L1-stage wild-type worms and the *wars-1* RNAi-treated worms with various concentrations of supplemental Trp and followed their development until adulthood. We found that at Trp concentrations higher than 10 mM, *wars-1* KD worms had developmental abnormalities, and nearly all were arrested before adulthood, while corresponding worms treated with control RNAi reached the adult stage (Fig. 7a).

Additionally, we aimed to determine the effect of acute Trp exposure in the germ cells and its impact on DNA damage response. We, therefore, treated the L4-stage wild-type worms with a high concentration of Trp for 24 h and stained the germline with the antibody against CHK-1p^Ser345^. In line with our previous data, treating the L4-stage worms with 100 mM of supplemental Trp activated the DNA damage response, confirmed by the increased nuclear staining of the activated CHK-1 (Fig. 7b).

### WARS-1 depletion or supplemental tryptophan increases intracellular levels of tryptophan and its catabolites

**Fig. 8:**
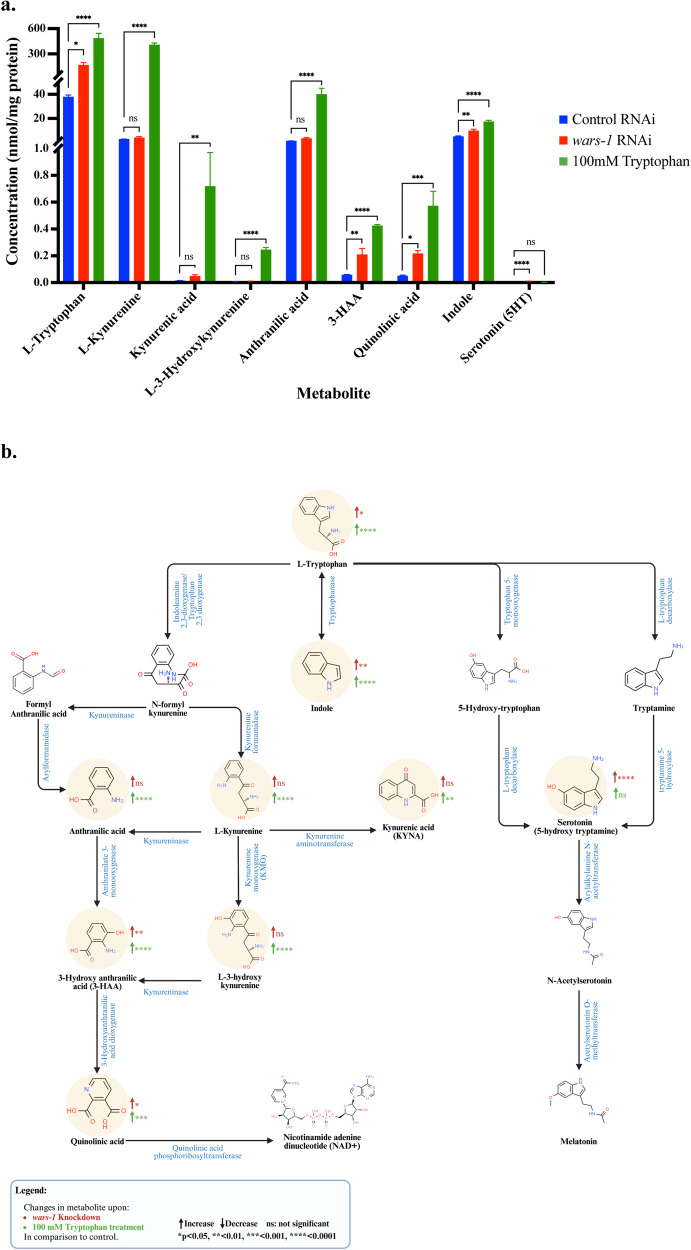
*wars-1* KD and high tryptophan supplementation lead to increased intracellular tryptophan and its catabolites. **a** Targeted metabolomics results. The graph shows the effects of *wars-*1 KD or treating worms with 100 mM Trp measured by targeted metabolomics. Targeted metabolomics showed significant changes in the level of L-tryptophan and eight of its catabolites. Measured metabolite concentrations were normalized to the corresponding protein content of each sample. **b** Simplified pathway of tryptophan metabolism. L-tryptophan is metabolized in the cells via three main pathways: kynurenine, indole, and serotonin pathways. Each metabolic pathway results in the production of distinct catabolites, each of which plays a crucial role in cellular functions. The detected metabolites in our targeted metabolomics are highlighted in yellow. The direction of change is shown in colored arrows (red for *wars-1* KD, green for 100 mM Trp), and stars show the level of significance (ns: not significant, *P < 0.05, **P < 0.01, ***P < 0.001, ****P < 0.0001). For each metabolite, ordinary one-way ANOVA was used to measure the significant differences between conditions. The Brown-Forsythe test was used to assess the equality of variances across groups and resulted in non-significant differences in the standard deviations (SDs) between conditions. The error bars represent the standard deviation (SD). For each condition (Control RNAi, *wars-1* RNAi, 100 mM Trp), three biological replicates, each extracted from 7000 adult worms, were analyzed.

Activation of the canonical DNA damage response upon exposure to 100 mM Trp (High-Trp) mimics the effect of *wars-1* KD. This data raises a dilemma of whether or not WARS-1 depletion leads to an increased level of intracellular tryptophan concentration, which, in turn, is cytotoxic, leading to the activation of the DNA damage response. To resolve such an important question, we used a targeted metabolomics approach to measure the level of Trp and its catabolites in both *wars-1* knockdown and wild-type worms. Interestingly, our results showed significant increases of 4-fold in the level of L-tryptophan upon *wars-1* KD (*P* = 0.0142) and 13-fold upon High-Trp (*P* < 0.0001, Fig. 8a, Sup. Figure S6a). Tryptophan is metabolized through tightly regulated pathways, each leading to the generation of various metabolites that play important roles in cell metabolism and survival. The Kynurenine pathway (KP) is the main route of Trp catabolism, mediated by tryptophan-2,3- dioxygenase-2 (TDO-2) in *C. elegans*, leading to the formation of L-Kynurenine, that can be further catabolized to other metabolites (Fig. 8b). Our metabolomics data shows a significant increase of L-kynurenine, Kynurenic acid, L-3-hydroxykynurenine, and anthranilic acid upon High-Trp (*P* < 0.0001, *P* = 0.0024, *P* < 0.0001, and *P* < 0.0001, respectively) but a non-significant increase upon *wars-1* KD (*P* = 0.9930, *P* = 0.9573, *P* = 0.8425, and *P* = 0.6829, respectively, Fig. 8a, Sup. Figure S6b-e). However, upon both High-Trp and *wars-1* KD, there was a significant increase in 3-hydroxy anthranilic acid (3-HAA) (*P* < 0.0001, *P* = 0.0002) and Quinolinic acid (*P* = 0.0011, *P* = 0.0453, Fig. 8a, Sup. Figure S6f, S3g). Other than the kynurenine pathway, Trp is metabolized through two other routes, forming other related metabolites (Fig. 8b). Both High-Trp supplementation and *wars-1* KD led to a significant increase in the level of Indole (*P* < 0.0001 and *P* = 0.0037, respectively, Fig. 8a, Sup. Figure S6h). Although the level of serotonin (5-hydroxy tryptamine) was significantly elevated upon *wars-1* KD, it was not affected upon treatment with the High-Trp (*P* < 0.0001, and *P* = 0.8919, respectively, Fig. 8a, Sup. Figure S6i).

Altogether, we showed that exposure to High-Trp mimics the effects of WARS-1 depletion in terms of increased Trp catabolites at a toxic level, leading to genomic instability. Also, the observed genomic instability upon *wars-1* KD is not a universal response to ARSs depletion or inhibition of translation, but rather it is specific to *wars-1* KD.

## Discussion

In this manuscript, we revealed a previously unknown role of WARS-1 protein in maintaining genome integrity. In summary, using cytological analysis and high-resolution imaging, we have demonstrated that *wars-1* KD in *C. elegans* resulted in a significant reduction of the mitotic index and CHK1 activation. The decreased mitotic activity was correlated with phosphorylated, inactive CDK1, consistent with a G2/M phase cell cycle arrest. Our cytological analysis showed intensive genomic instability upon WARS-1 depletion, leading to the formation of chromatin bridges, aberrant chromosomal structures associated with excessive DNA damage, or defects in DNA repair. Also, we showed micronuclei formation upon *wars-1* KD in the pachytene region of the germline and in developing embryos. We suggest that the depletion of WARS-1 or excess levels of Trp results in genomic instability, highlighting the importance of tryptophan-tRNA loading in preserving genome integrity. Importantly, by systematic depletion of all cytoplasmic ARSs or the inhibition of protein synthesis using CHX, we demonstrated the specificity of these findings to the WARS-1 depletion. Using targeted metabolomics, we showed a significant increase in the intracellular levels of Trp and its downstream metabolites upon WARS-1 depletion or High-Trp supplementation.

Using an antibody against H3p^Ser10^, we have shown that WARS-1 depletion reduces the mitotic index in the *C. elegans* germline. Phosphorylation of histone H3 at Ser10 is spatiotemporally correlated with chromatin condensation, initiating in G2 and peaking during the mitotic metaphase while significantly decreasing during the anaphase [27]. Such reduction in the mitotic index upon *wars-1* KD indicates either a proliferative function of the WARS-1 protein or mitotic cell cycle arrest in the absence of WARS-1. Cell cycle arrest is a normal physiological reaction to various stress conditions, the foremost of all occurring in response to DNA injury. It is well established that upon DNA damage in *C. elegans*, mitotically active germ cells stop cell cycle progression [34, 35]. One of the hallmarks of cell cycle arrest at the S phase of mitosis is the enlargement of the cell’s nuclei [36]. However, our observation revealed no significant differences in cell size between the mitotic cells in the germline of *wars-1* KD worms and the control, ruling out the possibility of S phase arrest.

Hyperphosphorylation of CDK-1 at Thr 14 and Tyr 15 is the canonical marker for the G2/M phase cell cycle arrest [37, 38]. During cell cycle progression, CDK-1 is generally dephosphorylated by phosphatases such as CDC25C to trigger mitosis entry, while upon genotoxic insult or due to incomplete replication, it is phosphorylated by upstream kinases such as checkpoint kinase 1/2 (CHK-1/2) [39]. Therefore, the phosphorylation status of CDK-1 is a great cytological marker to demarcate cells arrested at the G2/M phase transition. Our results showed a significant increase in CDK-1 phosphorylation in the mitotic cells of the germline, which strongly indicates that *wars-1* KD inhibits mitotic progression and leads to cell cycle arrest at the G2/M stage. CHK-1 phosphorylation at Serine 345, conferred by the upstream kinase ataxia telangiectasia and Rad3-related protein (ATR), is critical for checkpoint activation during the cell cycle [40]. *CHK1* activation hinders cell cycle progression and blocks G2/M phase transition and entry to mitosis upon DNA damage to prevent transmission of the damaged DNA to the daughter cells [41–43]. We observed an increase in CHK-1 activation at the mitotic zone and early, mid, and late pachytene stages of meiosis upon *wars-1* KD, indicating that WARS-1 depletion induced the classical checkpoint pathway most likely as a result of genomics instability and DNA damage response. This result is in accordance with the clinical data of cancer patients harboring mutations in *WARS1* [44].

More importantly, we observed the accumulation of chromatin-associated replication protein A (RPA-1) foci at the pachytene stage of meiotic cells in the germline, indicating excessive DNA damage. It is well established that the RPA-1 protein plays a vital role in various DNA metabolism, including DNA replication, meiotic recombination, and DNA repair. RPA-1 binds to single-stranded DNA (ssDNA), accumulating when DNA replication is perturbed, upon programmed double-strand breaks that are part of the normal meiotic events, or upon single and double-strand break processing. Also, the RPA-ssDNA complex is required for checkpoint signaling and activation of the ATM/ATR kinases pathway of the DNA damage response [45]. We, therefore, conclude that improper translation or an increase in the uncharged Trp enforced by the lack of aminoacyl-tRNA synthetase leads to the accumulation of DNA damage and activation of checkpoint signaling. However, we do not rule out the possibility that the RPA-1 accumulation and CHK-1 activation are related to unresolved recombination events that occur as part of normal meiotic events. Yet, we have clear evidence that WARS-1 depletion results in genomic aberration, which results in excessive formation of chromatin bridges, micronuclei, and aneuploidy in germ cells and developing embryos. Also, by knocking down all the members of the ARS family, we clearly showed that these effects are not seen in other ARSs KD.

In general, cytoplasmic micronuclei are usually formed by acentric chromosomal fragments, resulting in chromosomal loss during mitosis and meiosis cell division. Principally, aberrant chromosomal structures, such as micronuclei, fragmented chromatin, chromatin bridges, or buds, are canonical markers to trace mitotic catastrophes. Chromatin bridges, first described by Barbara McClintock in Maize upon exposure to X-ray, are formed due to the segregation of dicentric chromosomes during cell division [46]. Chromatin bridges form when dicentric chromosomes are segregated or when branched DNA intermediates fail to be processed. Chromatin bridges can rupture by mechanical means generated by actomyosin force or by nuclease cleavage involving TREX1 or alternatively be resolved by nucleases such as ANKLE1/LEM-3 in *C. elegans* [47–49]. Once chromatin bridges are broken, the free chromosome ends can fuse again and form additional bridges in the following cell cycle, entering the Breakage-Fusion-Bridge cycle (BFB), which leads to complex chromosomal rearrangements and genomics catastrophe [50].

We observed various-sized micronuclei structures in the *wars-1* RNAi germ cells comparable to mutants lacking double-strand processing abilities, such as MRE11, RAD50, and NBS1 mutants [51]. In both vertebrates and invertebrates, the MRE11 complex is essential to maintain genomic integrity during mitosis and the meiotic cell cycle by processing the site of DNA double-strand breaks generated by diverse endo or exogenous genotoxic events [52]. Although the accumulation of micronuclei and fragmented chromosomes upon *wars-1* RNAi are seen in the late stage of the meiotic prophase, they can potentially arise from catastrophic events that occurred earlier at the mitotic stage of the developing germline. These data align with the phosphorylation of CDK-1 in the mitotic germ cells upon *wars-1* KD. Formation of micronuclei and acentric chromosomal structures can lead to aneuploidy, which explains the defects in embryogenesis and sterility observed upon knocking down *wars-1*.

We also showed that while excessive Trp is toxic in wild-type worms, *wars-1* KD worms are hypersensitive to various Trp concentrations and are arrested during development. To our surprise, exposure of *C. elegans* to the acute dosage of Trp led to activation of the DNA damage response indicated by the phospho-activation of CHK-1. In essence, Trp is the least utilized amino acid by any given organism checked so far, and, more importantly, it is also the most infrequent amino acid that any given organism encounters during its life cycle. By exposing worms to increasing concentrations of Trp from their L1 stage, we observed developmental abnormalities in *wars-1* KD worms in concentrations higher than 10 mM, such that almost all were arrested before adulthood while control worms reached the adult stage in excess of Trp. This indicates that a physiological increase in Trp concentration has a cytotoxic effect upon *wars-1* KD during the development, to the extent that individual animals are developmentally arrested at various stages.

Surprisingly, Trp is also the least used in most of the DNA repair-related proteins and is predominantly enriched in the membrane-spanning proteins (Supl Fig. S1) [53]. This is explained by the complex structure of the Trp side chain, its overall hydrophobic nature, the presence of an indole ring for aquas interaction, and the cation-π interaction with positively charged amino acids such as Lysine and Arginine, which makes it an ideal moiety at bilayer interface [25, 26]. We hypothesize that upon knocking down *wars-1*, the cellular concentration of the unloaded Trp is increased to a toxic level, and the genomic instability observed is related to High-Trp and its metabolites rather than the improper translation. Therefore, using targeted metabolomics, we showed a significant increase in intracellular levels of Trp and some of its catabolites upon *wars-1* knockdown or High-Trp supplementation. Trp catabolism is a well-established immune system modulator in various types of cancer. It has been reported that an increase in Trp catabolism promotes tumor survival and metastasis [54, 55]. Tryptophan is degraded through the kynurenine pathway (KP) involving indoleamine 2,3 dioxygenase 1 and 2 (IDO1, IDO2), tryptophan 2,3 dioxygenase (TDO), and kynurenine monooxygenase (KMO) enzymes [56]. We showed that although the High-Trp supplementation significantly increased the concentration of kynurenine, kynurenic acid, hydroxy kynurenine, and anthranilic acid, WARS-1 depletion led to an increase, thus nonsignificant, in the levels of these metabolites. The concentration of 3-hydroxy anthranilic acid (3-HAA) and quinolinic acid (QA) have significantly increased upon WARS-1 depletion and supplementation with High-Trp. An increase in KP metabolites has been reported in various cancers, ranging from Leukemia to colorectal and lung cancer [57]. Accumulation of QA has been reported in human gliomas, and it has been linked to increased resistance to radiotherapy-induced oxidative stress and poor prognosis [58]. Also, increased QA phosphoribosyl transferase expression replenished the endogenous levels of nicotinamide adenine dinucleotide (NAD+) by converting QA to NAD+ and preventing apoptosis in malignant glioma cells [58]. Tryptophan is also catabolized to serotonin, an important neurotransmitter. Our metabolomics results showed a significant increase in the levels of serotonin upon *wars-1* KD but not after High-Trp supplementation. Although supplemental serotonin has long been known for its anti-depressant function, recent studies revealed its contribution to tumor migration, metastasis, and angiogenesis [59]. Cancer cells have evolved mechanisms to induce the synthesis of serotonin in favor of their proliferation and growth [60, 61]. Clinical studies of cancer patients’ metabolites classified cancer patients with active serotonin production as having poor prognosis [62].

Altogether, our data point out the genotoxic impact of *wars-1* knockdown, leading to genomic catastrophe and the formation of chromatin bridges. Notably, the acute exposure of wild-type worms to high concentration of Trp mimics the effect observed following *wars-1* RNAi, strongly suggesting that both elevated Trp levels and the depletion of tryptophanyl-tRNA synthetase, WARS-1, lead to activation of the canonical DNA damage checkpoint. This raises the question of how elevated Trp levels lead to genomic instability. Future studies are needed to dissect the effect of high Trp and/or its catabolites’ concentrations on genomic instability at the molecular level. All in all, our finding strongly suggests that WARS-1 is essential to keep the genome intact.

## Materials and Methods

### *C. elegans* strains and maintenance

*C. elegans* strains used in this study: Bristol *N2*, SSM473: *rpa-1(iow89[GFP11::rpa-1]) II; iowSi8 II; unc-119(ed3) III*. *C. elegans* strains were maintained at 20 °C on Nematode Growth Medium (NGM) plates seeded with *E. Coli* (OP50) bacteria, as described previously [63]. Worms at different developmental stages (embryo, Larval stages (L1, L2, L3, L4), and adults) were used and mentioned in the corresponding section. In this study, the hermaphrodite worms were used as we focused on the mitosis and meiosis events in the germline of *C. elegans*.

### Identifying tryptophan usage in the *C. elegans* proteome

To identify amino acid usage in *C. elegans* proteins, we have developed a Java-based program specifically designed to calculate the frequency of all the amino acids contributing to the *C. elegans* proteome. To accomplish this, we have generated a Hash table to store the data of all *C. elegans* protein sequences from the UniProt *C. elegans* reference proteome sequence (UP000001940). We measured each amino acid’s percentage in all *C. elegans* open reading frames relative to its total amino acid sequence, and the average percentage of each amino acid in the proteome of *C. elegans* was plotted.

### Bioinformatics and GO term analysis

Functional annotation and gene ontology (GO) enrichment analysis of proteins with more than twice the average (3%) or less than half the average (0.6%) tryptophan in their sequence was performed using the **D**atabase for **A**nnotation, **V**isualization, and **I**ntegrated **D**iscovery (DAVID) [64, 65]. Gene ID Conversion Tool was used to convert the UniProt IDs of a given protein set to a gene set. Terms enriched at the gene number count higher than 2 and EASE Score (a Modified Fisher Exact P value) equal to or less than 0.1 were considered significant. For GO terms of Cellular Component (CC), GOTERM_CC_DIRECT was selected. The GO terms resulting from DAVID were imported and visualized by the REVIGO (**RE**duce and **VI**sulize **G**ene **O**ntology) [66] and RStudio 2023.12.0 Build 369 “Ocean Storm” Release (R 4.3.2 GUI 1.80 Big Sur Intel build (8281)).

### ARSs RNAi construction

*C. elegans* total RNA was isolated using TRIzol reagent (15596026, Ambion, life technologies) and a Genejet RNA purification kit (K0731, Thermo Scientific), following the manufacturer’s recommendations. Isolated RNA was used to generate the *C. elegans* total cDNA library using a high-capacity cDNA reverse transcription kit (Cat # 4368814, Thermo Scientific). The coding sequence of *pars-1*, *kars-1*, *fars-1*, *yars-1*, *cars-1*, and *ears-1* were amplified using specific primers (Supl. Table S2). For *wars-1*, the standard protocol of NEBuilder Hifi DNA assembly (M5520AA, New England Biolabs (NEB)) was used to assemble the PCR products into the L4440 RNAi vector, while for other ARSs, the traditional cloning method was performed. The final product was transformed into the RNAi-expressing bacteria HT115(*DE3*).

### Knocking down ARSs using RNAi

All ARSs were knocked down using a homemade RNAi clone or an RNAi clone from the Ahringer *C. elegans* RNAi library (Source Bioscience) [67, 68].

### Inhibition of protein synthesis

To assess the effect of blocking protein translation, worms were treated with cycloheximide (CHX) (ab120093, Abcam) in liquid culture as previously described [30]. In brief, L4-stage worms were treated with a final concentration of 2 mg/ml CHX in M9 buffer for 6 h. Then, the germlines were extracted and immunostained using the specific antibodies.

### Western blotting

Western blotting was performed as previously described [69]. In summary, adult worms were lysed using bead-beating in the urea lysis buffer (7 M Urea, 100 mM DTT, 0.05% Triton, 25 mM NaCl, 20 mM HEPES). The soluble fraction was isolated by centrifugation at max speed for 30 min at 4 °C. Protein concentration was measured using the Pierce™ Bradford Plus Protein Assay Kit (23236, Thermo Scientific™, USA) following the manufacturer’s protocol. 10 µg of the protein samples were run on 4-12% gradient sodium dodecyl sulfate-polyacrylamide electrophoresis (SDS-PAGE) gels in MESS buffer at 200 volts for 35 min. Proteins were then transferred onto a polyvinyl difluoride (PVDF) membrane using the Trans-Blot® Turbo™ blotting System at 25 V for 30 min, and the membrane was blocked in the blocking solution (5% non-fat skim milk dissolved in PBS-tween (0.1%)) for 30 min at room temperature. After washing the membrane three times (each 10 min) with PBS-tween-20 (0.1%), it was incubated with primary rabbit antibody against WARS1 (PA5-29102, Thermo Scientific) (1:3000) and mouse anti-GAPDH antibody (MA1-16757, Thermo Scientific) (1:3000). HRP-conjugated anti-mouse (1:3000) and anti-rabbit (1:3000) secondary antibodies were used accordingly, and the markers were visualized using the ECL detection kit. Images were captured with an iBright™ FL1500 imaging system (Invitrogen, A44241). The western blot images were analyzed using the ImageJ software. The intensity of WARS-1 was measured and normalized to their corresponding band in the loading control (GAPDH).

### RNA isolation and Quantitative RT-PCR (qRT-PCR)

Adult worms were collected from plates and were washed 3 times with M9 buffer. Worms were then transferred into 1 ml of TRIzol™ Reagent (15596026, Invitrogen) and were frozen at −80 °C for at least 5 min. The frozen worms were lysed in the TRIzol reagent using 180 µm glass beads (G1152, Sigma-Aldrich) using a bead-beating apparatus (BEAD MILL 4, Fisher Scientific), and the total RNA was extracted following the manufacturer’s protocol. The total RNA was further purified using the RNeasy Mini kit (74104, Qiagen) according to the kit recommendations. *C. elegans* total cDNA was generated from 1 µg of purified RNA using the High-Capacity cDNA Reverse Transcription Kit (4374966, Applied Biosystems). qRT-PCR was performed by QuantStudio 6 Flex (Applied Biosystems) using the Power SYBR Green Master Mix (A25742, Applied Biosystems) in 20 µl reactions, each containing 4 µg of cDNA. Gene-specific primer sets were used to detect *wars-1* and *γ-tubulin* (Supl. Table S3). The cycle threshold (Ct) values for each target gene were extracted from the output file. The relative expression levels of the target genes were calculated using the ΔΔCt method by comparing the expression of the target gene (*wars-1*) to the reference gene (*γ-tubulin*).

### Immunostaining

Germline extraction and immunostaining were performed as described previously [69]. In brief, adult worms were transferred to the unseeded plates to crawl and eliminate the bacteria attached to their bodies. 8 to 10 worms were transferred into the dissection buffer (0.2 mM Levamisole, 0.2% Tween-20 dissolved in egg buffer) and added to a 22 × 22 mm coverslip. Worms were dissected near their pharynx or tail to extract the germline using a 23 Gauge syringe needle. The germlines were then fixed using fixation buffer (2% formaldehyde, 0.2 mM Tween-20 dissolved in egg buffer) and gently placed on a Poly-l-lysine coated slide. The slide was incubated for 5 min at room temperature and then flash-frozen in liquid nitrogen. Slides were removed from liquid nitrogen and freeze-cracked by instantly removing the coverslip and then incubated in ice-cold Acetone/Methanol (1:1, v-v) for 10 min. Slides were then treated with PBS-Triton x-100 (1% Triton x-100 in PBS) three times, each for 10 min, to increase the permeability to antibodies. Next, the slides were incubated in 1% Bovine Serum Albumin (BSA) (5217, TOCRIS) for 20 min to block the nonspecific binding of antibodies. Afterwards, the excess liquid was removed, and samples were treated with 40 µl of primary antibodies mixture working solution at 4 °C overnight. The next day, the slides were incubated in PBS-Tween-20 (0.1% Tween-20 in PBS) for 30 min, refreshing the solution every 10 min to decrease background staining. Next, the excess liquid was removed, and samples were incubated with 40 µl of secondary antibodies mixture working solution at room temperature for two hours. Afterward, they were washed again using PBS-Tween-20 (0.1% Tween-20 in PBS) for 30 min, refreshing the solution every 10 min. Finally, the slides were mounted using an antifade mounting media (H-1000, Vectashield) and 22 ×22 mm coverslips.

### Antibodies

The following primary antibodies were used with 1:200 concentrations: Anti-Phospho-CDK1 (Thr14, Tyr15) Mouse monoclonal Antibody (MABE229, EMD Millipore), Phospho-Histone H3 (Ser10) Polyclonal Antibody (PA5-17869, Thermo Scientific), Phospho-CHK1 (Ser345) Monoclonal Antibody (S.48.4) (MA5-15145, Thermo Scientific), Anti-alpha tubulin Mouse monoclonal [DM1A] antibody (ab7291, Abcam).

Secondary antibodies: Donkey Anti-Mouse IgG H&L (Alexa Fluor® 488) (Abcam, ab150105) (1:200), Donkey Anti-Rabbit IgG H&L (Alexa Fluor® 568) (ab175470, Abcam) (1:200), Donkey Anti-Mouse IgG H&L (Alexa Fluor® 647) (ab150107, Abcam) (1:200), Goat Anti-Rabbit IgG H&L (Alexa Fluor® 488) (ab150077, Abcam) (1:200). 0.5 μg/ml DAPI (diamidino-2-phenylindole) stain (62248, Thermo Scientific™) (1:200) was used to stain DNA.

### Microscopy and image analysis

The DM6 B Leica 3D-Thunder Imager Fluorescent microscope equipped with a C-mount Leica K5 (14401289) DOC TOP sCMOS Camera was used to visualize immunostained germlines and live worms. HC PL FUOTAR 40x/0.8 DRY, HC PL APO 63x/1.40 OIL, and HC PL APO 100x/1.40 OIL objective lenses were used. The Small Volume Computational Clearing (SVCC) method was used to remove out-of-focus blur and to reduce the background noise emission to further increase the clarity of the images. The z stacks (each ~0.3 μm) were used to export images and movies of different stacks or combined into a single image with maximum projection. For all the images, the digitalization was set to 16-bit. Microscopic images were captured using Leica Application Suite X (LAS X version 3.8.1.26810) software and analyzed using the same software and ImageJ (NIH) [70].

### Tryptophan hypersensitivity assay

To evaluate the effects of tryptophan supplementation on worms, NGM plates supplemented with final concentrations of 5 mM, 10 mM, 20 mM, and 30 mM of the amino acid L-tryptophan (T0254, Sigma-Aldrich) were prepared. Synchronized L1 worms were grown on the Trp-supplemented NGM plates seeded with control RNAi and *wars-1* RNAi. The hypersensitivity of worms to Trp was evaluated by scoring the percentage of animals that developed into adults, and worms were observed for nine consecutive days. We additionally prepared 30 mM, 40 mM, 50 mM, 80 mM, and 100 mM Trp-supplemented NGM plates to assess the effect of acute exposure to high Trp concentrations on checkpoint activity. Synchronized L4 stage worms were grown on normal and Trp-supplemented NGM plates until adulthood. Then, germlines were extracted from the day one adult worms and immunostained using CHK-1p^Ser345^, counterstained with DAPI to assess the effect of acute exposure to Trp on DNA damage response.

### Metabolite extraction

L1 worms were grown on *wars-1* RNAi or the control RNAi (HT115). To treat worms with a high concentration of Trp, the L4 stage worms grown on HT115 bacteria were transferred to NGM plates supplemented with 100 mM L-tryptophan (ab146400, Abcam). After 24 h of exposure, adult worms were collected from the plates and were washed 3 times with M9. Worms were then lysed using 200 mg of 180 µm glass beads (G1152, Sigma-Aldrich) and a bead-beating apparatus (BEAD MILL 4, Fisher Scientific) in 250 µl of water. Bead-beating was done six times, with each round lasting 30 s at maximum speed (5 m/s), with 20-second intervals on ice. To extract the metabolites, 700 µl of acetonitrile (34851, Honeywell) was added to each tube to have the final acetonitrile concentration of 70%. The bead-beating process was repeated six times, each lasting 30 s at maximum speed (5 m/s). Samples were then transferred to fresh 2 ml tubes and centrifuged at 21,000 g for 10 min at 10 degrees Celsius to pellet protein. The clear supernatants, which contain metabolites, were isolated. The protein pellets were used to measure the protein content using the Bradford assay, which was later used to normalize the metabolomics data.

### Targeted metabolomics for tryptophan and its catabolites

Nine-point serially diluted calibration solutions containing standard substances of all the targeted compounds were dissolved in an internal standard solution of isotope-labeled Trp and serotonin. 20 μL aliquots of extracted samples were dried in an N2 evaporator. To the dried residue, 20 μL of an internal standard solution was added. Next, 80 μL of dansyl chloride solution and 80 μL of a borate pH buffer were added to each sample or to a 20 μL aliquot of each calibration solution. The mixtures were incubated at 40 °C for 30 min. 10 μL aliquots of the resultant solutions were injected into a C18 column to run Liquid chromatography multiple reaction monitoring (MRM) mass spectrometry (LC-MRM/MS) on an Agilent 1290 liquid chromatograph coupled to a Sciex 7500 mass spectrometer with positive-ion detection, with the use of 0.1% formic acid in water and in acetonitrile isopropanol for binary-solvent gradient elution. For the assay of Trp, each sample solution was diluted 50-fold and then reinjected. 9-point serially diluted calibration solutions containing a standard substance of quinolinic acid were dissolved in 30% methanol. Each of the extractants was diluted 10 folds with 30% methanol. 10 μL aliquots of all the resultant solutions were injected into a 10-cm long C18 UPLC column to run LC-MRM/MS on an Agilent 1290 liquid chromatogram coupled to an Agilent 6495 C triple quadrupole mass spectrometer with negative-ion detection, with the use of an ammonium acetate solution and acetonitrile as the mobile phase for binary-solvent gradient elution. Concentrations of the detected analytes were calculated with internal or external standard calibration by interpolating their constructed linear regression curves with the data acquired from the sample solutions. Finally, the concentration of metabolites was normalized to their corresponding protein contents.

### Randomization and blinding

No randomization or blinding was used in the current study.

### Statistical analysis

Statistical analysis (Unpaired two-tailed t-tests with Welch’s correction and one-way ANOVA) and generating graphs were performed using GraphPad Prism 10 for macOS, Version 10.0.2 (171), GraphPad Software, Boston, Massachusetts, USA. The significance level was considered as P = 0.05. For all the experiments, at least 3 biological replicates were used to assess the statistical significance unless otherwise noted. When the t-test was performed, an F-test was also used to compare the variances for the groups being compared statistically. When ordinary one-way ANOVA was used to measure the significant differences between conditions, a Brown-Forsythe test was used to assess the equality of variances across groups. Statistical details of the experiments can be found in the results and discussion section and in the figure legends.

### Supplementary information

Supplementary information (Combined)

Original Data File

Supplemental Movie S1

Supplemental Movie S2

Supplemental Movie S3

Supplemental Movie S4

Supplemental Movie S5

Supplemental Movie S6

Supplemental Movie S7

Supplemental Movie S8

Supplemental Movie S9

Supplemental Movie S10

Supplemental Movie S11

Supplemental Movie S12

Supplemental Movie S13

Supplemental Movie S14

## Data Availability

All data generated and analyzed during the current study are available from the corresponding author on reasonable request.

## Code availability

The codes will be available upon request. Please contact the corresponding author.
