# Supplementary material for: Tryptophanyl-tRNA synthetase-1 (WARS-1) depletion and high tryptophan concentration lead to genomic instability in Caenorhabditis elegans
Source: Cell Death Discov. 2024 Apr 4;10:165. doi: 10.1038/s41420-024-01917-4 (PMC10995160; doi:10.1038/s41420-024-01917-4)
Supplement: Supplementary file 1 — Supplementary information (Combined) [file 41420_2024_1917_MOESM1_ESM.pdf]

List of Supplementary Materials

Supplemental Figure S1. TreeMap of Gene Ontology (GO) Cellular Component (CC) for tryptophan usage in *C. elegans* proteome.

Supplemental Figure S2. Confirmation of knocking down *wars-1*.

Supplemental Figure S3. Mitotic cell nuclei diameter.

Supplemental Figure S4. Assessing the effect of translational inhibition on cell cycle arrest, genomic instability, and DNA damage.

Supplemental Figure S5. Assessing the cell cycle arrest, genomic instability, and DNA damage in all other aminoacyl tRNA synthetase (ARSs).

Supplemental Figure S6. Targeted metabolomics results of individual metabolites.

Supplementary Table S1. The results of knocking down (KD) all 20 cytoplasmic aminoacyl tRNA synthetase using their corresponding RNAi.

Supplementary Table S2. List of primers used to amplify aminoacyl tRNA Synthetases (ARSs) coding sequence.

Supplemental Table S3. List of primers used in quantitative real-time PCR.

Supplemental Movie S1. Control RNAi $H3p^{Ser10}$ Staining (Red)

Supplemental Movie S2. Control RNAi Merge ($H3p^{Ser10}$(Red)-DAPI(Blue))

Supplemental Movie S3. Control RNAi Merge ($H3p^{Ser10}$(Red)-DAPI(Blue)-alpha-tubulin(Green))

Supplemental Movie S4. *wars-1* RNAi $H3p^{Ser10}$ Staining (Red)

Supplemental Movie S5. *wars-1* RNAi Merge (H3p^{Ser10}(Red)-DAPI(Blue))

Supplemental Movie S6. *wars-1* RNAi Merge (H3p^{Ser10}(Red)-DAPI(Blue)-alpha-tubulin(Green))

Supplemental Movie S7. Control RNAi CDK-1p$^{Thr14, Tyr15}$ Staining (Green)

Supplemental Movie S8. Control RNAi Merge (CDK-1p$^{Thr14, Tyr15}$(Green)-DAPI(Blue))

Supplemental Movie S9. *wars-1* RNAi CDK-1p$^{Thr14, Tyr15}$ Staining (Green)

Supplemental Movie S10. *wars-1* RNAi Merge (CDK-1p$^{Thr14, Tyr15}$(Green)-DAPI(Blue))

Supplemental Movie S11. Control RNAi CHK-1p^{Ser345} Staining (Red)

Supplemental Movie S12. Control RNAi Merge (CHK-1p^{Ser345}(Red)-DAPI(Blue))

Supplemental Movie S13. *wars-1* RNAi CHK-1p^{Ser345} Staining (Red)

Supplemental Movie S14. *wars-1* RNAi Merge (CHK-1p^{Ser345}(Red)-DAPI(Blue))

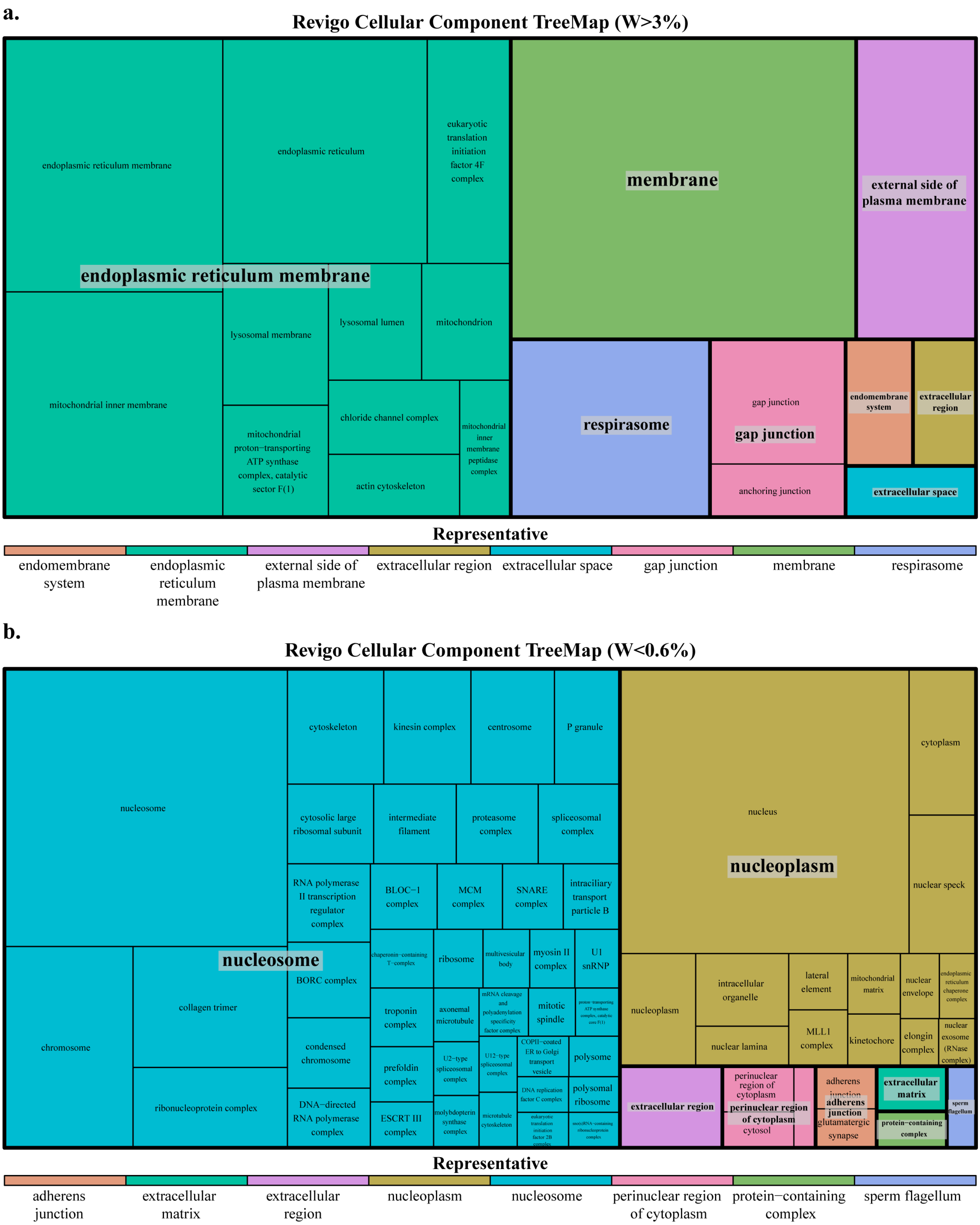

Supplemental Figure S1. TreeMap of Gene Ontology (GO) Cellular Component (CC) for tryptophan usage in *C. elegans* proteome. a. Revigo Cellular Component TreeMap (W>3%). The proteins with more than 3 percent tryptophan (W) in their sequence are mostly related to the membranes. **b. Revigo Cellular**

Component TreeMap (W<0.6%). The proteins with less than 0.6 percent Tryptophan in their sequence are mainly related to the nuclear components. The significantly enriched Gene Ontology (GO) terms were extracted from the DAVID database and visualized using REVIGO and R studio.

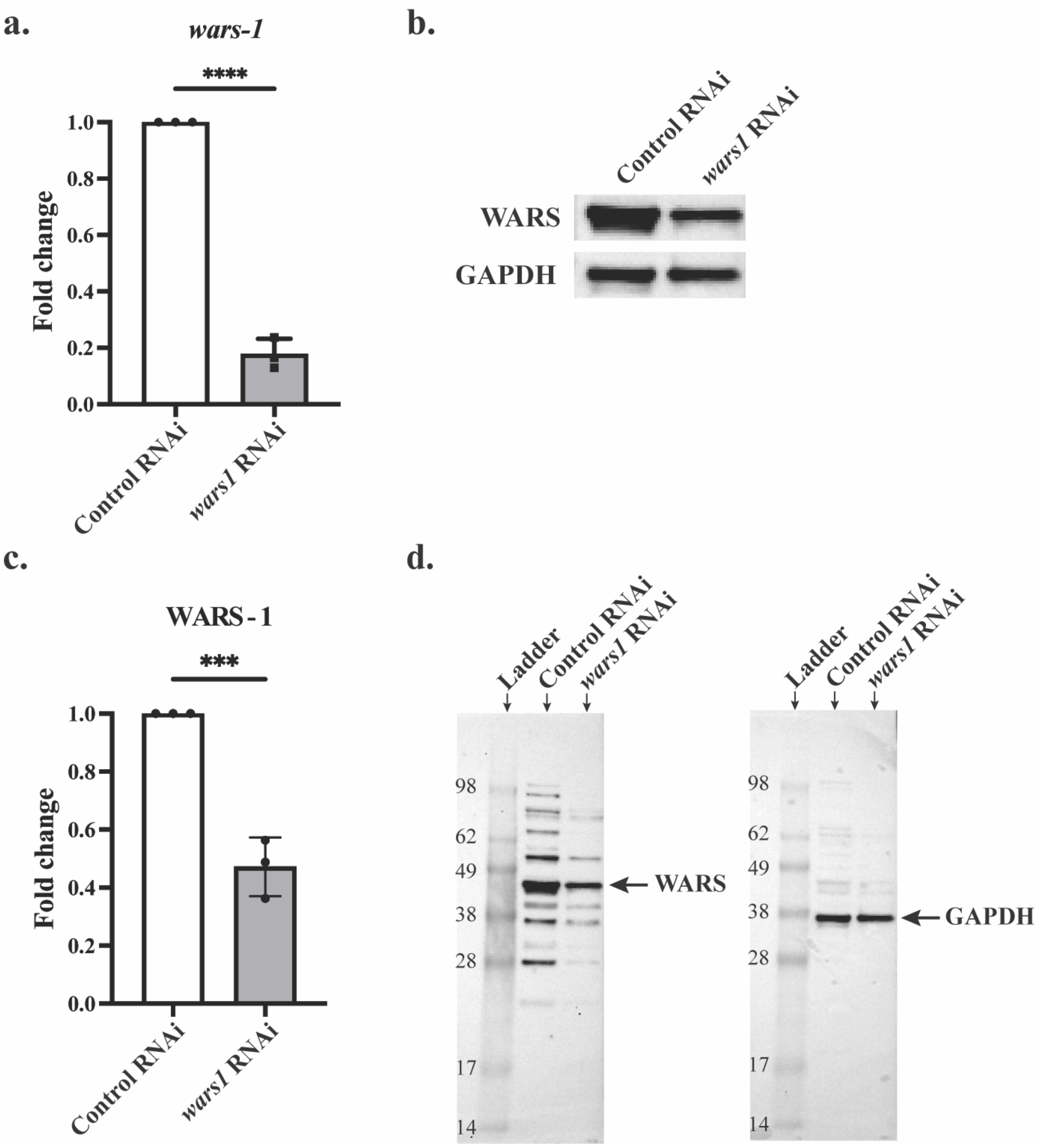

Supplemental Figure S2. Confirmation of knocking down *wars-1*. a. Confirmation using qRT-PCR. Knocking down *wars-1* using RNAi significantly reduced the expression of *wars-1* (**** $p<0.0001$). The experiment was repeated 3 times, and each time, it was performed in triplicates. The error bars represent the standard deviation. **b. Confirmation by Western blot.** *wars-1* knockdown led to a significant depletion of WARS-1 (N=3). The error bars represent the standard deviation. **c. The western blot** showed a 50% (~2-fold) decrease in the protein content. **d. Full gel images of representative Western Blot assay.** The sizes corresponding to each band of the ladder are shown on the left side of the gels (KD). Glyceraldehyde-3-phosphate dehydrogenase (GAPDH) was used as the loading control.

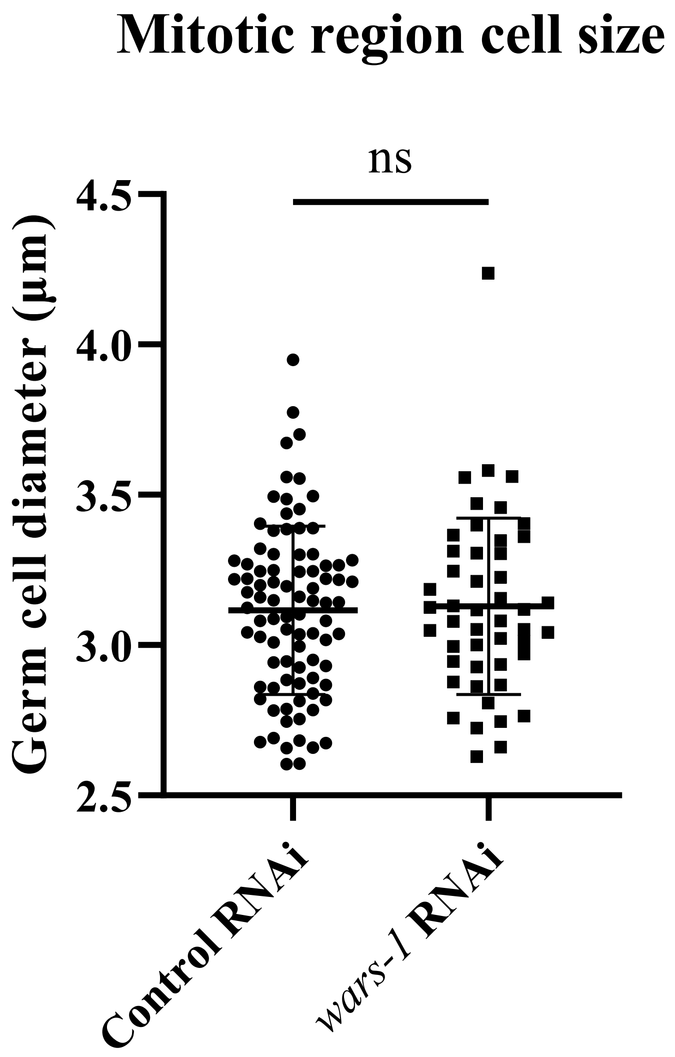

Supplemental Figure S3. Mitotic cell nuclei diameter.

Comparing the diameter of germ cells' nuclei in the mitotic region showed there is no significant difference between the cell size of mitotic cells in the *wars-1* Knock Down germline and control (P=0.802), ruling out the possibility of S phase arrest. For each condition, 3 biological replicates and at least 30 cells were measured. The error bar represents the standard deviation (SD).

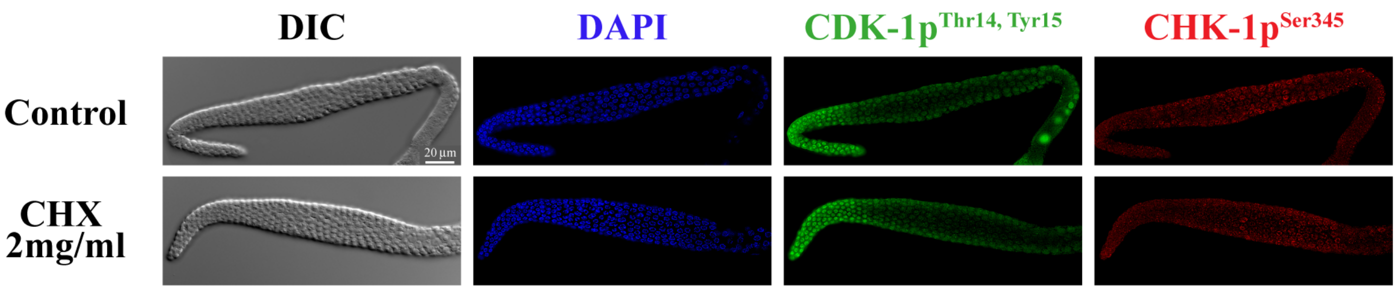

Supplemental Figure S4. Assessing the effect of translational inhibition on cell cycle arrest, genomic instability, and DNA damage. To check the effect of blocking protein synthesis on genomic instability, we performed chromatin staining using DAPI, which did not show any sign of genomic instability upon 2 mg/ml cycloheximide (CHX) treatment for 6 hours. Also, our analysis showed that there are no significant increases in the signal intensity of inactivated CDK-1 (CDK-1p$^{Thr14,\ Tyr15}$) staining compared to the control, indicating that there is no cell cycle arrest upon protein synthesis inhibition. Moreover, staining for phosphorylated checkpoint kinase-1 (CHK-1p^{Ser345}) ruled out the possibility of DNA damage induction upon blocking ribosomal protein translation. This experiment was performed on 3 biological replicates, and each time, more than 10 germlines were assessed. The scale bar shows 20 µm.

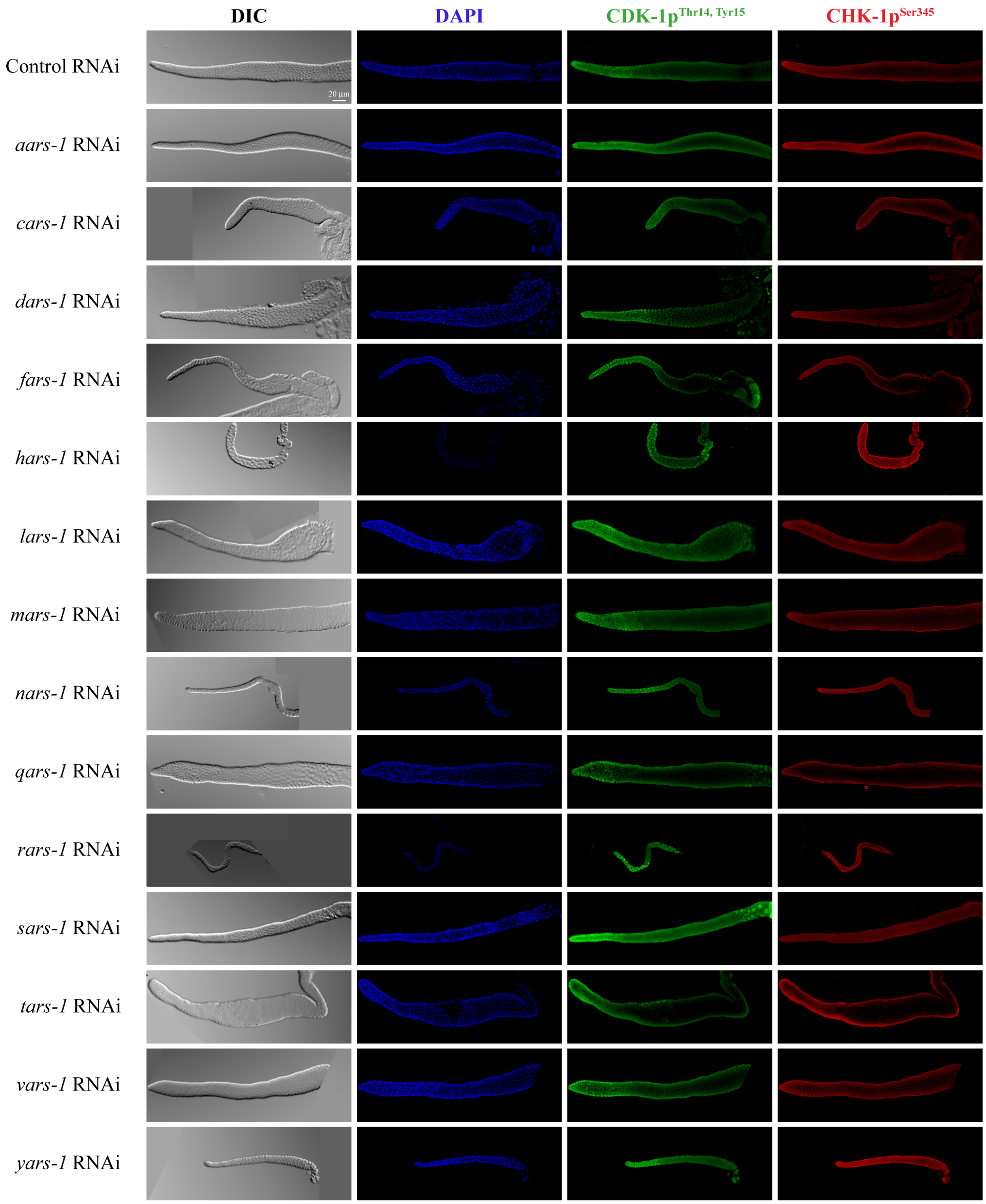

Supplemental Figure S5. Assessing the cell cycle arrest, genomic instability, and DNA damage in all other aminoacyl tRNA synthetase (ARSs). Our intensive chromatin staining using DAPI did not show any sign of genomic instability upon depletion of other ARSs. Also, our analysis of all cytoplasmic ARSs showed that there are no significant increases in the signal intensity of CDK-1p^{Thr14,Tyr15} staining compared to the control, indicating that there is no cell cycle arrest upon knocking down other cytoplasmic ARSs. Moreover, staining for phosphorylated checkpoint kinase-1 (CHK-1p^{Ser345}) failed to induce DNA damage upon knocking down all other ARSs. Depletion of GARS-1, IARS-1, KARS-1, EARS-1, and PARS-1 led to developmental arrest of the worms at different stages, which limited the possibility of germline staining. The scale bar shows 20 µm.

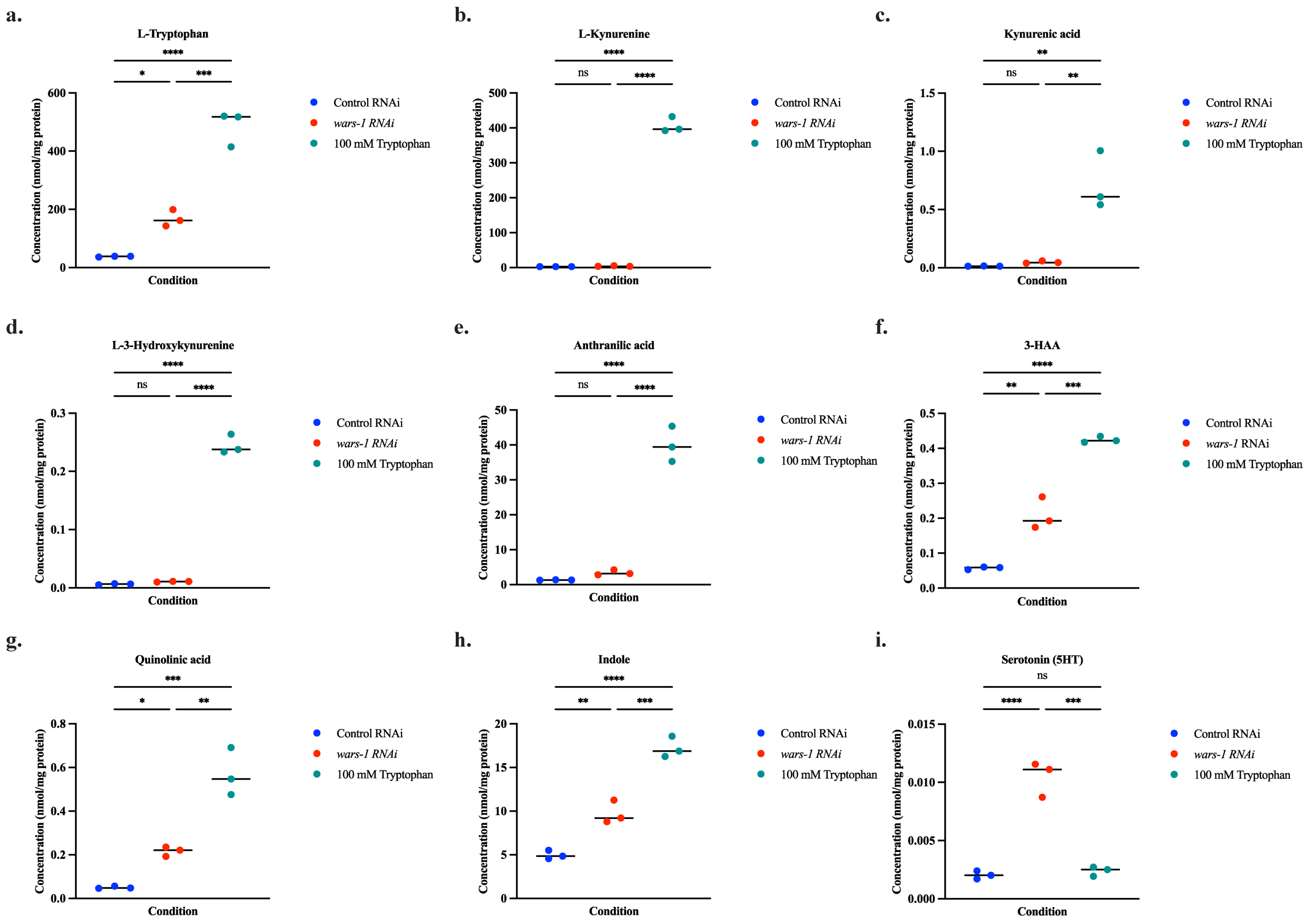

Supplemental Figure S6. Targeted metabolomics results of individual metabolites. a. L-Tryptophan. b. L-Kynurenine. c. Kynurenic acid. d. L-3-Hydroxykynurenine. e. Anthranilic acid. f. 3-hydroxy anthranilic acid (3-HAA). g. Quinolinic acid. h. Indole. i. Serotonin (5-hydroxy tryptamine (5-HT)). The error bars represent the standard deviation (SD). For each condition (Control RNAi, *wars-1* RNAi, 100 mM Tryptophan), 3 biological replicates, each extracted from 7,000 adult worms were analyzed.

Supplementary Table S1. The results of knocking down (KD) all 20 cytoplasmic aminoacyl tRNA synthetase using their corresponding RNAi.

Essential amino acids	KD Phenotype	Non-essential amino acid	KD Phenotype
**HARS-1**	100% sterile	**AARS-1**	Wild type
**IARS-1**	Developmentally delayed, 100% sterile	**RARS-1**	100% sterile
**LARS-1**	Same as wild type rather there are more embryos, lots of bag of worms	**NARS-1**	Wild type but delayed development
**KARS-1**	Developmentally delayed, 100% sterile	**DARS-1**	Almost wild type, small reduction in progeny number
**MARS-1**	Less progeny	**CARS-1**	100% sterile, dark inside
**FARS-1**	100% sterile	**EARS-1**	100% sterile, developmentally delayed, no germline
**TARS-1**	100% sterile	**QARS-1**	Wild type
**WARS-1**	100% sterile, fat accumulation	**GARS-1**	Adult but small size, destroyed germline, developmentally delayed, 100% Sterile
**VARS-1**	Reduced progeny, roller phenotype in some worms	**PARS-1**	Arrested at L3, 100% sterile
		**SARS-1**	100% sterile, looks adult but short
		**YARS-1**	100% sterile, dark inside

**Supplementary Table S2. List of primers used to amplify aminoacyl tRNA Synthetases (ARSs) coding sequence.**		
**ARSs**	**Primer Code**	**Primer Sequence**
wars-1	EP 300	5'-GCTCCAGGGGCGAATTGGGTACCGGGCCCATGGCAGCTCCAGCC GAACAAGTTG-3'
	EP 301	5'-GAATTCGAGCTCCACCGCGGTGGCGGCCGCATCTTGGAGCCACGT CACGTGTCATAC-3'
pars-1	EP 306	5'-accgggcccCTTACTTACAAGGAGGTTACCG-3'
	EP 307	5'-gtggcggccgcCCTCTACCATTGCATGCCAC-3'
kars-1	EP 308	5'-accgggcccCTGCTAAGCTGAATGTTGCCGTCG-3'
	EP 309	5'-gtggcggccgcGCTCCTCCAGCGATTTGGTTCATG-3'
fars-1	EP 310	5'-accgggcccATGACCGATCCGGATCGTCTCAACC-3'
	EP 311	5'-gtggcggccgcCTCTTCTTCAGCTCCTTCTTCTCATTTTCAC-3'
yars-1	EP 312	5'-ccgggcccccagGAATCACTTGGCGTTGATAAGC-3'
	EP 313	5'-gtggcggccgccttcgttgtccccgtacCCGG-3'
cars-1	EP 314	5'-ccgggcccGCTCAAGTCGGAAGTTCACGAGG-3'
	EP 315	5'-gtggcggccgcGAGCTTCGTACACCTTCTCCAGC-3'
ears-1	EP 316	5'-ccgggcccCGAGAAGAAGGGCGCCGTAATAAC-3'
	EP 317	5'-gtggcggccgcGGCGTTGGTCGATCCTGCAGC-3'

Supplementary Table S3. List of primers used in quantitative real-time PCR		
**Gene name**	**Primer Code**	**Primer Sequence**
wars-1	EP 405	5'-GGAAAGATATGAAGGTCGACGAG-3';
	EP 406	5'-CTCGGGCCTGATTCGTATTG-3'
γ-tubulin	EP 403	5'-AAGATCTATTGTTCTACCAGGC-3'
	EP 404	5'-CTTGAACTTCTTGTCCTTGAC-3'
