# Supplementary figures and images for: Tryptophanyl-tRNA synthetase-1 (WARS-1) depletion and high tryptophan concentration lead to genomic instability in Caenorhabditis elegans
Source: Cell Death Discov. 2024 Apr 4;10:165. doi: 10.1038/s41420-024-01917-4 (PMC10995160; doi:10.1038/s41420-024-01917-4)

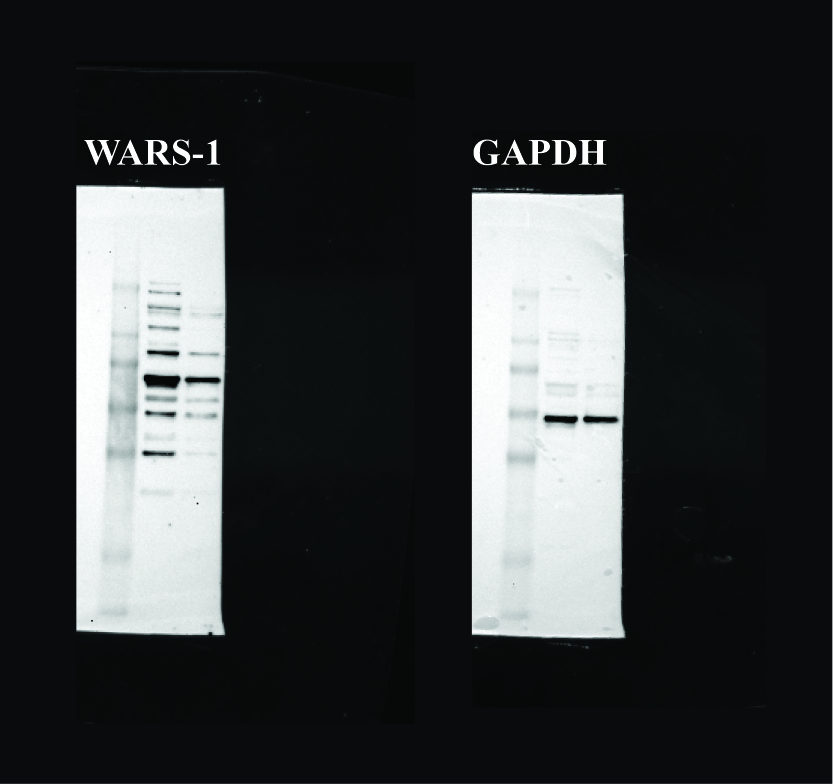

Supplement: Supplementary file 2 — Original Data File [file 41420_2024_1917_MOESM2_ESM.tif]
